# Une présentation inhabituelle de mycétome

**DOI:** 10.11604/pamj.2019.34.163.20300

**Published:** 2019-11-26

**Authors:** Edith-Sophie Bayonne Kombo

**Affiliations:** 1 Service de Dermatologie et Maladies Infectieuses, Hôpital de Référence de Talangaï, Brazzaville, Congo; 2 Faculté des Sciences de la Santé, Université Marien Ngouabi, Brazzaville, Congo

**Keywords:** Mycétome, tumeur cutanée, grains, Mycetoma, skin tumor, grains

## Abstract

Mycetoma is a chronic inflammatory process during which exogenous fungal or actinomycosic agents produce grains. The infection involves the skin, the subcutaneous tissues, the muscles and the bones. We report the case of a 39-year old woman, a teacher, living in Brazzaville, who had regularly resided in the village. She presented with a painless suppurative mass in the left thigh that had been treated with bandages over the past 4 years. The patient didn't have fever. Her general condition was good. Physical examination showed poly-lobed, firm tumor mass in the posterior surface of the left thigh, measuring 11cm in diameter on the major axis, with ulcerations discharging purulent serosity. There was no satellite adenopathy. The remainder of the physical examination was normal. Bacteriological analysis showed sterile serosity. Skin biopsy revealed alteration of the connective tissue and the presence of small foci of grains, typical of Actinomadura pelletieri. CRP level was 48mg/L; blood count, blood glucose, blood lipids and LDH were normal. Standard radiography of the affected thigh was normal. The diagnosis of actinomycosic mycetoma was retained. Patient's outcome, after a 12-month treatment with cotrimoxazole, was marked by the epidermization of the ulcers and the persistence of the tumor mass, justifying secondary surgical resection.

## Image en médecine

Le mycétome est un processus inflammatoire chronique au cours duquel des agents fongiques ou actinomycosiques d'origine exogène produisent des grains. L'infection affecte la peau, les tissus sous-cutanés, les muscles et les os. Nous rapportons le cas d'une femme de 39 ans, enseignante, habitant Brazzaville avec notion de séjours réguliers au village, qui avait consulté pour une masse suppurative indolore de la cuisse gauche, soignée par des pansements depuis 4 ans. Il n'y avait pas de fièvre. Son état général était conservé. L'examen physique notait une masse tumorale polylobée, siégeant sur la face postérieure de la cuisse gauche, ferme, mesurant 11cm de diamètre dans son grand axe, surmontée d'ulcérations laissant soudre une sérosité purulente. Il n'y avait pas d'adénopathies satellites. Le reste de l'examen était normal. La sérosité était stérile à l'analyse bactériologique. La biopsie cutanée révélait une altération du tissu conjonctif et la présence de petits foyers de grains caractéristiques d'actinomadura pelletieri. La CRP était à 48mg/l; l'hémogramme, la glycémie, les lipides sanguins et les LDH étaient normaux. La radiographie standard de la cuisse affectée était normale. Le diagnostic de mycétome actinomycosique était retenu. L'évolution, après un traitement de cotrimoxazole pendant 12 mois, était marquée par l'épidermisation des ulcérations et la persistance de la masse tumorale justifiant une exérèse chirurgicale dans un second temps.

**Figure 1:**
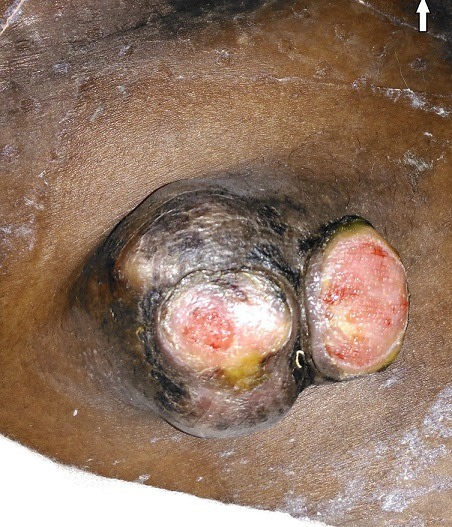
Mycétome de la cuisse

